# The impact of modifiable risk factor reduction on childhood asthma development

**DOI:** 10.1186/s40169-018-0195-4

**Published:** 2018-06-11

**Authors:** Andrew Abreo, Tebeb Gebretsadik, Cosby A. Stone, Tina V. Hartert

**Affiliations:** 1 Department of Medicine, Center for Asthma Research, Vanderbilt University Medical Center, Nashville, TN USA; 2 Department of Biostatistics, Center for Asthma Research, Vanderbilt University Medical Center, Nashville, TN USA

**Keywords:** Asthma, Risk factors, Pediatrics

## Abstract

Childhood asthma is responsible for significant morbidity and health care expenditures in the United States. The incidence of asthma is greatest in early childhood, and the prevalence is projected to continue rising in the absence of prevention and intervention measures. The prevention of asthma will likely require a multifaceted intervention strategy; however, few randomized controlled trials have assessed such approaches. The purpose of this review was to use previous meta-analyses to identify the most impactful risk factors for asthma development and evaluate the effect of risk factor reduction on future childhood asthma prevalence. Common and modifiable risk factors with large effects included acute viral respiratory infections, antibiotic use, birth by cesarean section, nutritional disorders (overweight, obesity), second hand smoke exposure, allergen sensitization, breastfeeding, and sufficient prenatal vitamin D level. Evaluation and estimates of risk factor modification on populations at risk should guide scientists and policymakers toward high impact areas that are apt for additional study and intervention.

**Electronic supplementary material:**

The online version of this article (10.1186/s40169-018-0195-4) contains supplementary material, which is available to authorized users.

## Introduction

Asthma is the leading chronic disease among children, affecting 8.4% of children in the United States and up to 14% worldwide [1, 2]. The economic impact and loss of productivity are enormous compared to other childhood illnesses [3]. The overall prevalence of asthma in the US has been increasing over the past 30 years in all demographic groups [4]. A similar increase in prevalence is noted throughout the developing world, although the association with economic development is not uniform [5]. Currently there are no demonstrated effective interventions for the primary prevention of asthma [6].

The rise in asthma and allergy prevalence is happening at a rate that cannot be fully explained by genetic factors. Although a positive family history is a highly predictive risk factor, its positive predictive value for the development of childhood asthma only ranges from 11 to 37%. The marked increase in prevalence over a short time period suggests that multiple environmental risk factors are driving an upward trend in prevalence that cannot be explained by genetic factors alone [7]. In utero or early life risk factors for asthma include maternal health and stress, mode of delivery, exposure to environmental pollutants and allergens, obesity, diet, and exposure to infections and antibiotics, among others. The risk of developing childhood asthma likely depends on a combination of host factors and their interaction with a multitude of environmental factors rather than one single risk factor; however, data on the differential and combined impact of risk factors is lacking [8].

Finland faced a similar public health crisis in 1972 when confronted with very high cardiovascular disease mortality rates. Similar to asthma, the development of cardiovascular disease requires the interaction of multiple genetic and environmental factors, including tobacco use, obesity, physical inactivity, hypercholesterolemia, hypertension, and diabetes. Leaders of the North Karelia Project targeted multiple risk factors and implemented policies on a community and national level, including anti-smoking legislation and strict school meal nutritional requirements. The cardiovascular mortality rate decreased by 65% over the next 2 decades, demonstrating that population-based public health interventions are an effective strategy for prevention of a gene by environment disease [9].

The prevention of asthma will likely require a similar multi-faceted approach, targeting multiple relevant risk factors to prevent disease. The aim of this review is to estimate the impact of modifying risk factor exposure on future childhood asthma development with population level estimates to examine the policy implications of these projections. This paper is the first to use pre-existing data to identify the most impactful risk factors of childhood asthma, and to estimate the effect of risk factor reduction on disease prevalence.

## Methods

### Search strategy and inclusion criteria

We conducted a search of the PubMed database from 1990 to October 2017 to identify meta-analyses evaluating the association between individual risk factors and asthma development. We used the search term “asthma” and restricted the results to meta-analyses, children or adolescents (0–18 years), and articles published in English. A second search was completed and limited to meta-analyses and female gender to capture maternal pregnancy risk factors. Inclusion of abstracts identified by the initial screen was based on specific criteria: (1) published by a peer-reviewed journal; (2) provided a point estimate such as odds ratio with corresponding 95% confidence intervals (CI); and (3) outcomes were assessed in children or adolescents. The outcome of interest was the risk of developing incident asthma in childhood. For risk factors with more than one meta-analysis available for review, we selected either the most recent analysis or that with the largest study population. The characteristics derived from each selected article included the type of risk factor, study authors, year of publication, size of the study population, definition of asthma diagnosis, and odds ratio estimates with corresponding 95% CI.

### Prevalence of risk factors

The prevalence of each risk factor in the US population was identified through a comprehensive literature and database review. Risk factor prevalence estimates were restricted to an age group window for which data was available, and reflect the age ranges in the meta-analyses that we used. The four age exposure windows were prenatal, 0–1, < 5, and 5–13 years. In the absence of risk factor prevalence data for a specific exposure window, prevalence estimates for children 0–18 years of age were applied to the 5–13 years group. The impacted population size of each risk factor was calculated using 2015 Centers for Disease Control National Vital Statistics System (NVSS) birth data and 2010 US Census data [10, 11]. The total number of live births from the 2015 NVSS was used to calculate the impacted population size for prenatal and infant risk factors.

### Data analysis: population attributable fraction

The contribution of a specific risk factor to the burden of asthma in the population was quantified using population attributable fraction (PAF). PAF is a useful public health tool because it measures the proportion of cases that could be prevented by eliminating a risk factor through control measures in a target population. The calculation of PAF assumes a causal relationship between a risk factor and a disease; however, asthma is a multifactorial disease likely caused by multiple additive factors [12]. A similar methodology has been used to estimate the impact of risk factor reduction on the prevalence of Alzheimer’s disease and cardiovascular disease [13, 14]. A formula proposed by Levin in 1953 was used to calculate the PAF for each risk factor:

$${\text{PAF}} = \left[ {{{{\text{P}}_{\text{RF}} *\left( {{\text{OR}} - 1} \right)} \mathord{\left/ {\vphantom {{{\text{P}}_{\text{RF}} *\left( {{\text{OR}} - 1} \right)} {\left( {1 + {\text{P}}_{\text{RF}} *\left[ {{\text{OR}} - 1} \right]} \right)}}} \right. \kern-0pt} {\left( {1 + {\text{P}}_{\text{RF}} *\left[ {{\text{OR}} - 1} \right]} \right)}}} \right]$$

Levin’s formula relies on the prevalence of risk factor exposure in the population (P_RF_) and the strength of the association between a risk factor and disease (OR, odds ratio) [15]. Although the original formula included relative risk as a measure of association, we used odds ratios as an estimate of relative risk based on the available data in the studies meeting criteria for inclusion.

The total number of cases attributable to each risk factor was obtained by multiplying the PAF for each risk factor by the total number of incident asthma cases in children ages 5–11. This age group was selected for two reasons. First, the onset of asthma occurs before 8 years of age in 80% of cases [16]. Second, the best available asthma prevalence data for children under 8 years is reflected by the prevalence of asthma in the United States between the ages of 5 and 11–9.6% or approximately 2.76 million children [1]. We then projected the impact of a 10 and 25% reduction in the prevalence of individual risk factors on the total number of attributable cases by re-calculating the PAF for each risk factor using reduced prevalence estimates.

## Results

**Fig. 1:**
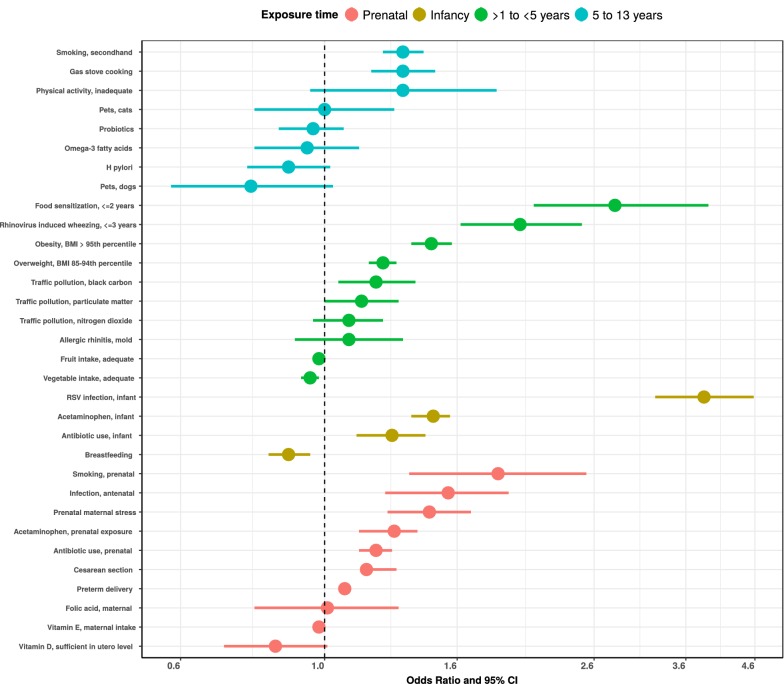
Association of risk factors for asthma development within age exposure windows. The four age exposure windows are prenatal, 0–1, < 5, and 5–13 years. Odds ratios and 95% confidence intervals are shown ranked by effect size and directionality. References used to determine point estimates for this figure: smoking, secondhand [69]; gas stove cooking [96]; physical activity, inadequate [64]; pets, cats [57]; probiotics [97]; omega-3 fatty acids [98]; *H. pylori* [99]; pets, dogs [57]; food sensitization, ≤ 2 years [77]; rhinovirus induced wheezing, ≤ 3 years [24]; obesity [65]; overweight [65]; traffic pollution [100]; allergic rhinitis, mold [101]; fruit intake, adequate [102]; vegetable intake, adequate [102]; RSV infection, infant [23]; acetaminophen, infant [103]; antibiotic use, infant [45]; breastfeeding [88]; smoking, prenatal [70]; infection, antenatal [104]; prenatal maternal stress [105]; acetaminophen, prenatal [103]; antibiotic use, prenatal [44]; cesarean section [37]; preterm delivery [106]; folic acid, maternal [107]; vitamin E, maternal intake [92]; vitamin D, sufficient in utero level [108]

**Fig. 2:**
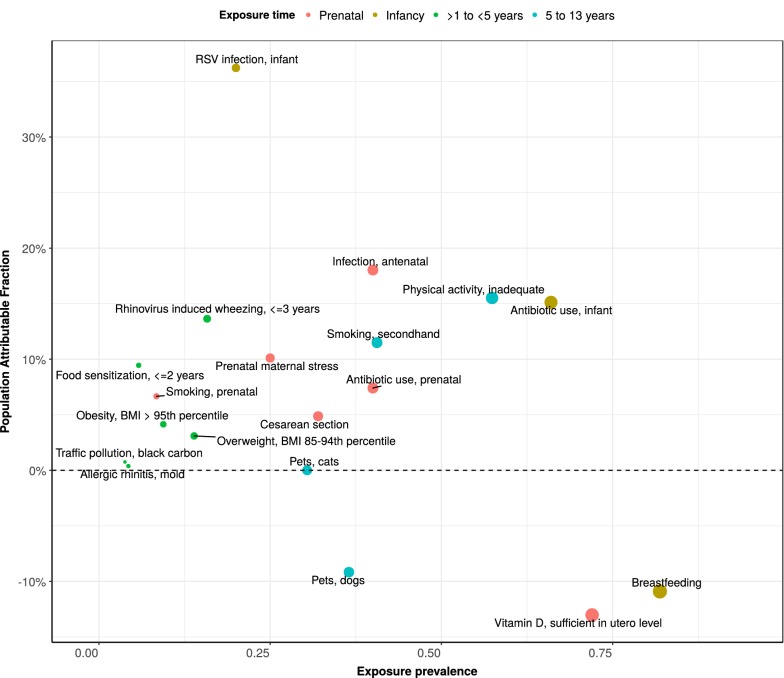
Population attributable fraction (PAF) among selected risk factors. PAF, the proportion of cases that are attributable to a risk factor and could be prevented by modifying or eliminating the risk factor, is shown on the y-axis. The x-axis represents the risk factor exposure prevalence. The points on the graph reflect the relationship of exposure prevalence and PAF. The PAF is dependent on the prevalence of exposure and its odds ratio for asthma. The size of the points are proportional to prevalence. Odds ratios from meta-analyses were used to estimate PAF (Additional file 1: Table S1 and Fig. 1). References used to determine exposure prevalence for this figure: smoking, secondhand [71]; gas stove cooking [109]; physical activity, inadequate [110]; pets, cats [111]; probiotics [112]; omega-3 fatty acids [112]; *H. pylori* [113]; pets, dogs [111]; food sensitization, ≤ 2 years [114]; rhinovirus induced wheezing, ≤ 3 years [29, 30]; obesity [61]; overweight [115]; traffic pollution [116]; allergic rhinitis, mold [117]; fruit intake, adequate [118]; vegetable intake, adequate [118]; RSV infection, infant [28]; acetaminophen, infant [119]; antibiotic use, infant [42]; breastfeeding [87]; smoking, prenatal [72]; infection, antenatal [41]; prenatal maternal stress [120]; acetaminophen, prenatal [121]; antibiotic use, prenatal [41]; cesarean section [72]; preterm delivery [72]; folic acid, maternal [122]; vitamin E, maternal intake [123]; vitamin D, sufficient in utero level [124]

**Fig. 3:**
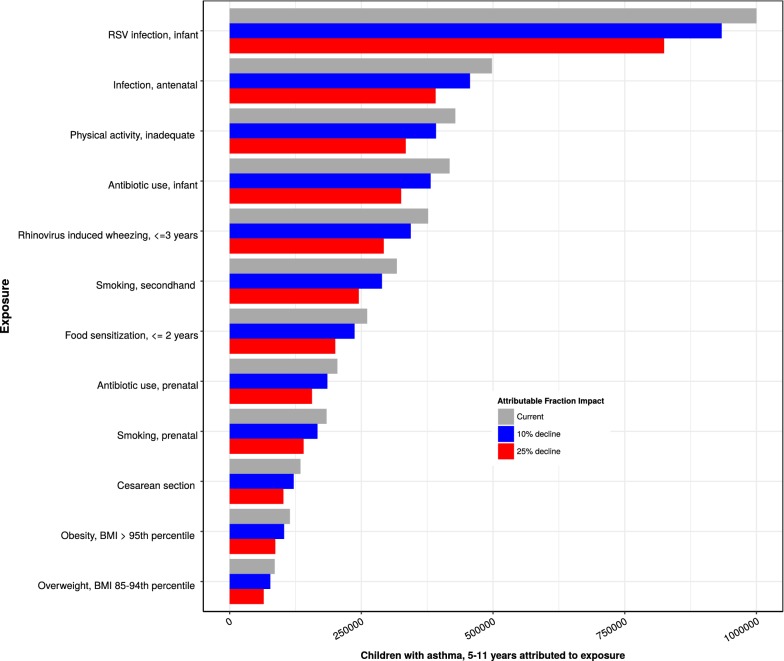
Impact of reducing selected risk factor exposure on asthma cases among children 5–11 years of age. Based on an asthma prevalence of 9.6% from the Centers for Disease Control and Prevention to estimate 2,761,000 children with asthma. Twelve risk factors were selected for ranking based on their effect size, prevalence of exposure, and potential for modification. This figure considers only the main effect of the risk factor (without accounting for potential interactions)

We identified thirty-two meta-analyses examining the association between individual risk factors and asthma development. Additional file 1: Table S1 presents the odds ratios and prevalence data for each risk factor used in the analysis. Figure 1 illustrates the strength and direction of each associated risk factor on asthma development by age exposure window and descending order of effect size. The risk factors with the strongest association, based on the size of the odds ratio, were prenatal maternal smoking, infant RSV infection, food allergen sensitization in early childhood, secondhand smoke exposure, and physical inactivity in late childhood. However, these odds ratios do not take into account the prevalence of risk factor exposure and do not reflect the population attributable fraction (PAF). Figure 2 uses PAF and exposure prevalence to illustrate the impact of each modifiable risk factor on asthma development in the population. PAF provides an indication of the potential percent reduction in incidence of asthma in a given population if the exposure is causal and is eliminated. There is no pre-specified way of evaluating the importance of PAF. Therefore, the magnitude of the PAF and exposure prevalence were evaluated relative to other risk factors in the same age exposure window. Risk factors with a high PAF, high exposure prevalence, and high modifiable impact are the most important public health targets. These risk factors include acute viral respiratory infections, antibiotic use, birth by cesarean section, nutritional disorders (overweight, obesity), second hand smoke exposure, and allergen sensitization. Equally important are risk factors with a protective effect such as breastfeeding and sufficient maternal vitamin D levels. Figure 3 shows the effect of a 10 and 25% reduction in specific, modifiable risk factors on the estimated number of children between 5 and 11 years of age with asthma attributable to the risk factor. Assuming each risk factor plays an independent role, approximately 51% of asthma cases are attributable to two exposures that occur during infancy (RSV lower respiratory tract infection and antibiotic use).

## Discussion

The greatest incidence of asthma development occurs during early childhood [17]. The risk of asthma onset is likely influenced by gene-environment interactions. The goal of primary prevention is to prevent the development of disease by identifying and reducing modifiable risk factors within the environment [18]. Given the complexity in identifying candidate genes that predict who will develop asthma, preventive strategies should focus on primary prevention of prenatal and early childhood risk factors with a high population attributable fraction. However, there are a limited number of randomized controlled trials assessing the efficacy of such interventions on asthma incidence. The Canadian Asthma Primary Prevention Study (CAPPS), one of the only multifaceted intervention trials in asthma, showed that multiple interventions before birth and through the 1st year of life could reduce the prevalence of childhood asthma by 7 years of age [19]. Our study is the first to use pre-existing study results to identify the most impactful risk factors for childhood asthma, and to estimate the effect of potential targeted intervention strategies on disease prevalence.

Based on the effect size as a measure of association with outcome, prevalence of exposure, and potential for modification, the following risk factors were found to have the largest impact on childhood asthma development: acute viral respiratory infections, antibiotics, birth by cesarean section, nutritional disorders (overweight, obesity), second hand smoke exposure, and allergen sensitization. These will be discussed below.

### Acute viral respiratory infections

Acute respiratory infections (ARI) are the leading cause for ambulatory visits and hospitalizations in infants [20, 21]. Respiratory syncytial virus (RSV) and human rhinovirus (HRV) are the most commonly detected viruses during infant ARIs. RSV is the primary etiology of infant lower respiratory tract infection (LRTI) that may require hospitalization in severe cases while HRV may cause LRTI, but more often leads to a mild upper respiratory infection [22]. In addition to the increased burden in the acute care setting, both RSV and HRV lower respiratory tract infections are strongly associated with the development of early childhood wheezing and asthma [23, 24]. However, it is unclear whether the association is the result of a genetic predisposition or directly attributable to the virus. Two previous studies found that the administration of RSV immunoprophylaxis to preterm infants was associated with a reduction in recurrent wheezing, suggesting a potential causal role of RSV LRTI in the development of asthma [25, 26]. Similar observational or intervention studies do not yet exist to support a definitive relationship associating HRV with asthma [27].

Our analysis indicates that the largest proportion of childhood asthma in the United States is attributable to RSV infection during infancy. Both RSV and HRV are highly prevalent and potentially modifiable risk factors in early childhood. The prevalence of RSV LRTI is 20% in the 1st year of life, whereas the prevalence of HRV-induced wheezing is 15.8% in the first 3 years of life [28–30]. Current strategies to prevent RSV infection include avoidance of exposure, reduction of tobacco smoke exposure, birth timing, and passive immunization with RSV immunoprophylaxis. RSV immunoprophylaxis is effective in reducing morbidity in infants; however, the high total cost and burden of administration limit its use to high-risk children. While there is strong evidence that RSV is a causal factor in asthma development, whether prevention of RSV can significantly reduce the risk of developing asthma is a question best addressed by a randomized controlled trial [31]. Future research should also be directed toward the prevention of RSV through active immunization of infants and/or pregnant women and study of the longer term effects on childhood asthma development. The implementation of influenza and tetanus-diphtheria-acellular pertussis vaccination programs for pregnant women is a successful model for the protection of both the mother and infant [32]. There are currently 10 candidate RSV vaccines in ongoing clinical trials, including 3 maternal vaccines and 7 pediatric vaccines [33]. The development of active and passive immunization strategies against HRV lags behind RSV, and is limited by the sheer number of viral serotypes and lack of cross-protective immunity between serotypes [34].

### Perinatal events

The cesarean birth rate has increased from 4.5% in 1965 to 32% in 2015 without a significant improvement in maternal or infant outcomes over the past 10 years [35, 36]. Based on a recent meta-analysis, infants born via cesarean section had an approximately 20% increase in the onset of asthma [37]. These children are also at higher risk for other chronic diseases such as diabetes and obesity, so a reduction in exposure should result in multiple health benefits [38]. The American College of Obstetricians and Gynecologists led the charge to decrease cesarean delivery in 2013 by recommending against elective cesarean delivery prior to 39 weeks of gestation due to the risk of complications [39]. National organizations, health care systems, and individual providers could also re-evaluate current delivery practices to reduce the cesarean birth rate. In addition, all health care systems could proactively release metrics on deliveries to enhance transparency and enable data-driven quality improvement, as is required of hospitals accredited by The Joint Commission [40].

Equally alarming is the rising rate of antibiotic use in the United States. Antibiotic use during pregnancy is reported in 40% of women and in 66% of infants in the 1st year of life [41, 42]. The Centers for Disease Control and Prevention (CDC) reports that at least 30% of all outpatient antibiotics are incorrectly prescribed for the management of viral infections. Inappropriate antibiotic use increases the risk of short-term and long-term complications, including alteration of the infant microbiome [43]. The relative risk of asthma development is increased by 20% in infants exposed to antibiotics in utero and 27% in infants exposed to antibiotics within the 1st year with a very strong dose response relationship [44–46]. Similar to cesarean delivery, infant and prenatal antibiotic use is associated with a multitude of chronic health conditions including inflammatory bowel disease, obesity, and diabetes [47]. All members of the health care team have a role in antibiotic stewardship. Health care systems should provide adequate education and feedback to providers. Providers should use strategies such as watchful waiting and frequent reassessment of need, and patients should be fully engaged in the decision making process [43].

One theory suggests that the effect of cesarean delivery and antibiotics on the development of the immune system is due to alteration of the microbiome. The human microbiome is a collection of symbiotic microorganisms located in the skin, respiratory tract, gastrointestinal tract, and genitourinary tract. Commensal bacteria contribute to human health by performing essential metabolic functions and modulating the emerging immune system [48]. Dysbiosis, or a derangement in the composition of commensal bacteria, can be induced by several environmental factors including cesarean birth, antibiotic use, and dietary changes [49]. The infant microbiome varies predictably depending on the mode of delivery, and recent studies suggest that vaginal microbial transfer may partially restore the microbiome of cesarean-born infants [50, 51]. Another potential mechanism of asthma development due to cesarean delivery is alteration of early air-lung exchange by retention of amniotic fluid in the infant lungs [52]. Pre- and post-natal antibiotic exposure is similarly associated with reduced diversity of the newborn intestinal flora [53]. The clinical relevance of dysbiosis is illustrated by the hygiene hypothesis, which postulates that a lack of early life exposure to a microbe rich environment favors the development of asthma [54]. Growing up in a microbe rich environment, such as a farm, appears to be protective against developing asthma; however, there is no meta-analysis for inclusion into this paper and randomized controlled trials are ongoing [55]. A current clinical trial aims to determine whether administration of an oral bacterial extract to high risk infants can prevent the development of wheezing respiratory illness and asthma [56]. Another microbe rich environment includes pet ownership. A recent meta-analysis failed to find a strong association between pet ownership and a reduction in asthma risk; however, there are studies that suggest a protective effect of dogs [57–59].

### Obesity

Childhood obesity is a modern public health crisis. In children, obesity is defined as a body mass index (BMI) greater than the 95th percentile for age and gender [60]. The prevalence of obesity among children < 5 years of age has doubled from 4.8 to 9.4% over the past 40 years [61]. This increase is most likely due to a combination of complex factors including physical inactivity, altered dietary patterns, and other environmental exposures. Moreover, childhood obesity contributes to the development of asthma, sleep apnea, diabetes mellitus, and cardiovascular disease [62]. The mechanism linking obesity and asthma is poorly understood, but likely related to the pro-inflammatory effects of adipose tissue [63]. Other drivers of obesity, including physical inactivity and overweight status, also have a similar impact on asthma development [64, 65].

Body mass index is the recommended method of screening for obesity beginning at 2 years of age. Current management focuses on mitigating disease severity through secondary and tertiary prevention strategies such as dietary and lifestyle modifications. Pediatricians can play an important role by recommending increased physical activity, avoidance of sweetened beverages, and elimination or reduction of television exposure [66]. However, public health campaigns must also ensure that all children have access to affordable, nutritious food and after school programs that allow at least 60 min of daily exercise in a safe environment. In addition, local school systems should consider implementing multifaceted school-based programs given their effectiveness in the primary prevention of childhood obesity. However, these programs require significant involvement of both parents and educators and may be less cost-effective than other interventions such as lifestyle modifications [67, 68].

### Second hand smoke exposure

Pre- and post-natal exposure to tobacco smoke has long been recognized as a risk factor for the development of asthma. The risk of asthma onset is increased by 85% in infants exposed to maternal smoking during pregnancy and by 32% in children exposed to secondhand smoke [69, 70]. These findings are especially troubling given the prevalence of tobacco smoke exposure is 8.4% prenatally and 40.6% between 5 and 13 years of age [71, 72]. Screening caregivers for the use of tobacco products is an obligation of all medical providers [73]. Current therapeutic recommendations focus on proven strategies such as cessation counseling and pharmacotherapy; however, additional policies and funding are needed to increase access and affordability of these services. Reducing the overall prevalence of tobacco use will likely require increased regulation by policymakers such as implementing more robust smoke-free laws, raising cigarette list prices or taxes, and further limiting tobacco advertisements [74]. Parental smoking is a modifiable predictor of smoking initiation during adolescence, so preventing parental smoking is also of critical importance to the long-term behavior of children [75].

### Allergen sensitization

The rise of food allergy over the past 20 years parallels the increasing prevalence of childhood asthma in the United States [76]. Recent studies show that food allergen sensitization prior to 2 years of age is a significant risk factor for the development of asthma; however, it is unclear whether this is an independent risk factor or another disease in the atopic march [77]. The recent increase in food allergy may be attributable to previous guidelines that recommended delayed introduction of allergenic foods until after the 1st year of life [78]. More recently, the Learning Early About Peanut Allergy (LEAP) study found that the early introduction of peanut between 4 and 11 months of age in low- and moderate-risk children reduced the onset of peanut allergy [79]. Pediatricians can play a critical role in the prevention of food allergy. The National Institute of Allergy and Infectious Disease recommends early introduction of peanut-containing foods between 4 and 6 months of age to high-risk infants after evaluation for allergy testing and around 6 months for infants with mild-to-moderate eczema [80]. There are ongoing trials studying the efficacy of oral, sublingual, and epicutaneous immunotherapy in the primary prevention of peanut allergy; however, no specific therapy is on the market and it is unknown whether there will be an impact on asthma development [81].

Early sensitization to perennial aeroallergens is associated with an increased risk of asthma development [82]. Exposure to house dust mite (HDM) has been shown to be an important predictor of childhood asthma in multiple studies [83, 84]. Two multifaceted intervention trials that included HDM avoidance were associated with a decreased prevalence of asthma; however, it is less clear whether HDM avoidance alone would have the same effect [19, 85]. In addition, an ongoing clinical trial aims to evaluate whether blocking IgE with omalizumab can prevent progression to asthma in preschool children with wheezing respiratory illness and aeroallergen sensitization [86]. There are no current recommendations for strategies to prevent aeroallergen sensitization; however, patients should be educated on avoidance techniques for seasonal and perennial allergens once diagnosed.

### Protective factors

Our review revealed two factors that may be protective against the development of childhood asthma: breastfeeding and sufficient prenatal vitamin D level. These are cost-effective, easily implemented interventions that could offer potential benefit with low risk. Breastfeeding rates vary by duration with 81.9% of infants initiating at birth, 60.6% up to 6 months, and 34.1% up to 1 year [87]. Breastmilk provides early passive immunity through the biologic activity of immunoglobulins; however, the mechanism for protection from asthma and other allergic disorders is unclear [88]. Based on guidelines from the American Academy of Pediatrics, providers should strongly recommend exclusive breastfeeding for at least 6 months with mixed feeding including breastmilk through 12 months [89].

Vitamin D levels are insufficient in approximately 28% of pregnant women between 12 and 44 years of age [90]. Multiple studies suggest a protective effect of higher levels of in utero 25-hydroxy-vitamin D against the development of asthma. Combined analysis of two recent randomized controlled trials showed that prenatal vitamin D supplementation may reduce the risk of childhood asthma [91]. Providers should ensure that women of childbearing age are taking routine prenatal vitamins; however, the need for additional vitamin D supplementation to prevent asthma has yet to be determined. Increased maternal intake of vitamin E (alpha-tocopherol) during pregnancy is associated with protection against early childhood wheezing, but has not been definitively shown to protect against the development of childhood asthma [92]. More than 90% of adults in the United States who do not use supplements fail to meet recommended intake of vitamin E and D, but could with supplementation [93].

## Limitations

Several assumptions were made to perform this analysis. First, the use of population attributable fraction (PAF) assumes a causal relationship between risk factor exposure and the development of asthma. Asthma, similar to cardiovascular disease, is likely the result of multiple, interacting risk factors. PAF estimates the proportion of disease that could be eliminated if the risk factor is removed; however, the absence of causality diminishes its value. We have previously reviewed the available levels of causal evidence for many of these risk factors [94, 95]. We are not implying causality is a settled matter for all of the discussed risk factors; however, our intent was to guide policymakers and scientists using a novel analytic plan. Second, the robustness of the PAF calculation is dependent on the strength of the meta-analysis and accuracy of the risk factor prevalence data. Prevalence data can vary based on the population being assessed. We limited our analysis to childhood risk factors for which there was a meta-analysis in order to improve statistical precision. We chose to focus on childhood risk factors because most children experience asthma symptoms by 6 years of age. However, this exclusion criterion eliminated many potentially modifiable risk factors for which no meta-analysis exists. For each risk factor, we selected the meta-analysis that was either the most recent or had the largest study population, knowing that each study may be limited by methodology and subject to confounding. A detailed assessment of each meta-analysis for confounding was not completed given the study was designed to highlight key areas and opportunities for targeted studies to support causal relationships and public health interventions. Therefore, the results should be interpreted with caution given the extent of confounding or reverse causation may vary depending on the risk factor. For example, the association between inadequate physical activity and asthma may be due to exercise limitation from poor symptom control, an example of reverse causation. Prenatal risk factors, such as antibiotic use, cesarean section, and maternal stress, are not subject to reverse causation. However, socioeconomic status as a potential confounder may not have been controlled for in individual studies within a meta-analysis. The upward bias of meta-analyses is an important limitation in using this type of data; however, use of summary data from meta-analyses is a means of incorporating a summary effect size of the available published studies. We are less concerned about an upward bias related to RSV infection because it meets many of the Bradford Hill criteria for causality, compared with a number of risk factors for which there have not been studies conducted to fully support causal relationships. Third, the Levin formula uses unadjusted relative risk to calculate PAF; however, we used odds ratio given it was consistently available across all the included meta-analyses. Odds ratio may overestimate the risk ratio when an outcome is common in a study population. Fourth, risk factor prevalence estimates were based on the best available and most recent data. We were unable to calculate age-standardized rates; therefore, estimates were sorted into the age group for which data was available. This allowed for comparison of PAF within each exposure window since PAF estimates do not take into account the size of the population affected by a risk factor. The impacted population size for prenatal and infant risk factors was calculated using the total number of live births rather than the total number of pregnancies given the focus was on the development of asthma in living children. Fifth, while we provide impact estimates of relevant risk factors based on their strong association and high prevalence, these estimates do not replace the necessity of randomized controlled trials to determine the effect of risk factor modification on future asthma prevalence. Finally, we understand that there are many issues that are likely to affect the success of primary prevention efforts, including a better understanding of the phenotypes of asthma, selecting target risk populations for exposure specific interventions, heterogeneity in treatment effects, mediating effects, etc. For a large list of risk factors, it is not possible to include all of this evidence in a single review. Therefore, we chose to focus on what we believe is the most important in informing the next needed steps in the field—a focus on risk factors that are prevalent, modifiable, and have a large effect size, and to present the data on their potential impact on asthma incidence using PAF. Regarding the overall contribution of PAF, the magnitude of the impact of asthma as a lifelong chronic disease is sufficiently large that even a small reduction in true asthma incidence would have major health implications. Our hope is that the novel compilation of data will inform and prioritize the field toward studies to confirm causality and to conduct public health interventions for primary disease prevention.

## Conclusion

In the United States, a significant proportion of childhood asthma may be attributable to modifiable risk factors including acute viral respiratory infections, antibiotic use, birth by cesarean section, obesity, second hand smoke exposure, and allergen sensitization. Breastfeeding and sufficient prenatal vitamin D concentrations may be protective against asthma onset. In the absence of effective primary prevention strategies, current management focuses on reducing the impairment and risk associated with asthma. However, this study shows that multifactorial prevention of early childhood risk factors could reduce the future prevalence of asthma. Additional randomized controlled trials are needed to provide evidence of causality of the risk factor-disease relationship and to identify effective preventive strategies. Causal evidence and controlled trials of known asthma risk factors will be essential in driving policymakers to create more effective community-based programs and public health strategies targeting high prevalence risk factors with high population attributable fraction.

## Additional file

**Additional file 1: Table S1.** Meta-analyses and exposure prevalence data of risk factors for childhood asthma development.

## Abbreviations

CI: confidence interval
NVSS: National Vital Statistics System
PAF: population attributable fraction
P_RF_: prevalence of risk factor exposure in the population
OR: odds ratio
CAPPS: Canadian Asthma Primary Prevention Study
ARI: acute respiratory infection
RSV: respiratory syncytial virus
HRV: human rhinovirus
LRTI: lower respiratory tract infection
CDC: Centers for Disease Control and Prevention
BMI: body mass index
LEAP: Learning Early About Peanut Allergy
HDM: house dust mite
