# Supplementary material for: The impact of modifiable risk factor reduction on childhood asthma development
Source: Clin Transl Med. 2018 Jun 11;7:15. doi: 10.1186/s40169-018-0195-4 (PMC5995769; doi:10.1186/s40169-018-0195-4)
Supplement: Supplementary file 1 — Additional file 1: Table S1. Meta-analyses and exposure prevalence data of risk factors for childhood asthma development. [file 40169_2018_195_MOESM1_ESM.docx]

| **Table S1:**  **Meta-analyses and Exposure Prevalence Data of Risk Factors for Childhood Asthma Development** | | | |
| --- | --- | --- | --- |
| **Risk Factor (Citation)** | **Study Population**  **(total patients)** | **Odds Ratio**  **(95% confidence interval)** | **Risk Factor Prevalence** |
| **Prenatal (3,978,497 live births)** |  |  |  |
| Acetaminophen, prenatal [1, 2] | 24 studies: 425,140 | 1.28 (1.13-1.39) | 65.5% |
| Antibiotic use, prenatal [3, 4] | 10 studies: 2,118,978 | 1.20 (1.13-1.27) | 40% |
| Cesarean section [5, 6] | 26 studies: 3,390,258 | 1.16 (1.14-1.29) | 32% |
| Folic acid, maternal [7, 8] | 5 studies: 45,642 | 1.01 (0.78-1.30) | 40% |
| Infection, antenatal [4, 9] | 10 studies: 299,830 | 1.55 (1.24-1.92) | 40% |
| Prenatal maternal stress [10, 11] | 10 studies: 3,210,204 | 1.45 (1.25-1.68) | 25% |
| Preterm delivery [6, 12] | 19 studies: 456,651 | 1.074 (1.072-1.075) | 9.63% |
| Smoking, prenatal [6, 13] | 5 studies: 23,857 | 1.85 (1.35-2.53) | 8.4% |
| Vitamin D, sufficient in utero level [14, 15] | 10 studies: 8,871 | 0.84 (0.70-1.01) | 72% |
| Vitamin E, maternal intake [16, 17] | 6 studies: 787,742 | 0.98 (0.96-0.99) | 19.8% |
| **Age 0 – 1 (3,978,497 live births)** |  |  |  |
| Acetaminophen, infant [1, 18] | 24 studies: 425,140 | 1.47 (1.36-1.56) | 87.8% |
| Antibiotic use, infant [19, 20] | 18 studies: 336,225 | 1.27 (1.12-1.43) | 66% |
| Breastfeeding , ever [21, 22] | 13 studies: 341,684 | 0.88 (0.82-0.95) | 81.9% |
| RSV infection, infant [23, 24] | 15 studies: 82,008 | 3.84 (3.23-4.58) | 20% |
| **Age < 5 (20,201,362 children)** |  |  |  |
| Allergic rhinitis, mold [25, 26] | 8 studies: 31,742 | 1.09 (0.9-1.32) | 4.3% |
| Food sensitization, ≤ 2 years [27, 28] | 7 studies: 6,996 | 2.8 (2.1-3.9) | 5.8% |
| Fruit intake, adequate [29, 30] | 41 studies: 162,715 | 0.98 (0.96-1.00) | 49.8% |
| Obesity, BMI > 95^th^ percentile [31, 32] | 38 studies: 1,411,335 | 1.46 (1.36-1.57) | 9.4% |
| Overweight, BMI 85-94^th^ percentile [31, 33] | 38 studies: 1,411,335 | 1.23 (1.17-1.29) | 13.9% |
| Rhinovirus induced wheezing, ≤ 3 years [34-36] | 15 studies: 2,004 | 2.00 (1.62-2.49) | 15.8% |
| Traffic pollution, black carbon [37, 38] | 11 studies: 63,944 | 1.20 (1.05-1.38) | 3.8% |
| Traffic pollution, nitrogen dioxide [37, 38] | 11 studies: 63,944 | 1.09 (0.96-1.23) | 3.8% |
| Traffic pollution, particulate matter [37, 38] | 11 studies: 63,944 | 1.14 (1.00-1.30) | 3.8% |
| Vegetable intake, adequate [29, 30] | 41 studies: 162,715 | 0.95 (0.92-0.98) | 21.7% |
| **Age 5 – 13 (36,859,869 children)** |  |  |  |
| Gas stove cooking [39, 40] | 19 studies: 72,387 | 1.32 (1.18-1.48) | 9.3% |
| H pylori [41, 42] | 24 studies: 50,290 | 0.88 (0.76-1.02) | 16.7% |
| Omega-3 fatty acids [43, 44] | 8 studies: 6,541 | 0.94 (0.78-1.13) | 1.1% |
| Pets, cats [45, 46] | 10 studies: 11,489 | 1.00 (0.78-1.28) | 30.4% |
| Pets, dogs [45, 46] | 9 studies: 11,433 | 0.77 (0.58-1.03) | 36.5% |
| Physical activity, inadequate [47, 48] | 11 studies: 47,807 | 1.32 (0.95-1.84) | 57.4% |
| Probiotics [44, 49] | 14 studies: 3,143 | 0.96 (0.85-1.07) | 0.5% |
| Smoking, secondhand [50, 51] | 20 studies: 225,245 | 1.32 (123-1.42) | 40.6% |

**Additional References**

1. Etminan M, Sadatsafavi M, Jafari S, Doyle-Waters M, Aminzadeh K, FitzGerald JM. Acetaminophen use and the risk of asthma in children and adults: a systematic review and metaanalysis. Chest. 2009;136(5):1316-23.

2. Werler MM, Mitchell AA, Hernandez-Diaz S, Honein MA. Use of over-the-counter medications during pregnancy. Am J Obstet Gynecol. 2005;193(3 Pt 1):771-7.

3. Zhao D, Su H, Cheng J, Wang X, Xie M, Li K, et al. Prenatal antibiotic use and risk of childhood wheeze/asthma: A meta-analysis. Pediatr Allergy Immunol. 2015;26(8):756-64.

4. Ledger WJ, Blaser MJ. Are we using too many antibiotics during pregnancy? BJOG. 2013;120(12):1450-2.

5. Huang L, Chen Q, Zhao Y, Wang W, Fang F, Bao Y. Is elective cesarean section associated with a higher risk of asthma? A meta-analysis. J Asthma. 2015;52(1):16-25.

6. Curtin SC, Abma JC, Ventura SJ, Henshaw SK. Pregnancy rates for U.S. women continue to drop. NCHS Data Brief. 2013(136):1-8.

7. Crider KS, Cordero AM, Qi YP, Mulinare J, Dowling NF, Berry RJ. Prenatal folic acid and risk of asthma in children: a systematic review and meta-analysis. Am J Clin Nutr. 2013;98(5):1272-81.

8. Daniels K, Daugherty J, Jones J. Current contraceptive status among women aged 15-44: United States, 2011-2013. NCHS Data Brief. 2014(173):1-8.

9. Zhu T, Zhang L, Qu Y, Mu D. Meta-analysis of antenatal infection and risk of asthma and eczema. Medicine (Baltimore). 2016;95(35):e4671.

10. van de Loo KF, van Gelder MM, Roukema J, Roeleveld N, Merkus PJ, Verhaak CM. Prenatal maternal psychological stress and childhood asthma and wheezing: a meta-analysis. Eur Respir J. 2016;47(1):133-46.

11. Yali AM, Lobel M. Coping and distress in pregnancy: an investigation of medically high risk women. J Psychosom Obstet Gynaecol. 1999;20(1):39-52.

12. Jaakkola JJ, Ahmed P, Ieromnimon A, Goepfert P, Laiou E, Quansah R, et al. Preterm delivery and asthma: a systematic review and meta-analysis. J Allergy Clin Immunol. 2006;118(4):823-30.

13. Burke H, Leonardi-Bee J, Hashim A, Pine-Abata H, Chen Y, Cook DG, et al. Prenatal and passive smoke exposure and incidence of asthma and wheeze: systematic review and meta-analysis. Pediatrics. 2012;129(4):735-44.

14. Feng H, Xun P, Pike K, Wills AK, Chawes BL, Bisgaard H, et al. In utero exposure to 25-hydroxyvitamin D and risk of childhood asthma, wheeze, and respiratory tract infections: A meta-analysis of birth cohort studies. J Allergy Clin Immunol. 2017;139(5):1508-17.

15. Mansbach JM, Ginde AA, Camargo CA, Jr. Serum 25-hydroxyvitamin D levels among US children aged 1 to 11 years: do children need more vitamin D? Pediatrics. 2009;124(5):1404-10.

16. Wu H, Zhang C, Wang Y, Li Y. Does vitamin E prevent asthma or wheeze in children: A systematic review and meta-analysis. Paediatr Respir Rev. 2017.

17. Kim HJ, Giovannucci E, Rosner B, Willett WC, Cho E. Longitudinal and secular trends in dietary supplement use: Nurses' Health Study and Health Professionals Follow-Up Study, 1986-2006. J Acad Nutr Diet. 2014;114(3):436-43.

18. Lundgren M, Steed LJ, Tamura R, Jonsdottir B, Gesualdo P, Crouch C, et al. Analgesic antipyretic use among young children in the TEDDY study: no association with islet autoimmunity. BMC Pediatr. 2017;17(1):127.

19. Penders J, Kummeling I, Thijs C. Infant antibiotic use and wheeze and asthma risk: a systematic review and meta-analysis. Eur Respir J. 2011;38(2):295-302.

20. Kinlaw AC, Sturmer T, Lund JL, Pedersen L, Kappelman MD, Daniels JL, et al. Trends in Antibiotic Use by Birth Season and Birth Year. Pediatrics. 2017;140(3).

21. Lodge CJ, Tan DJ, Lau MX, Dai X, Tham R, Lowe AJ, et al. Breastfeeding and asthma and allergies: a systematic review and meta-analysis. Acta Paediatr. 2015;104(467):38-53.

22. Centers for Disease Control and Prevention. Breastfeeding Report Card. 2016. https://www.cdc.gov/breastfeeding/pdf/2016breastfeedingreportcard.pdf. Accessed 28 December 2017.

23. Regnier SA, Huels J. Association between respiratory syncytial virus hospitalizations in infants and respiratory sequelae: systematic review and meta-analysis. Pediatr Infect Dis J. 2013;32(8):820-6.

24. American Academy of Pediatrics. Respiratory Syncytial Virus. In: Kimberlin DW, Brady MT, Jackson MA, Long SS, editors. Red Book: 2015 Report of the Committee on Infectious Diseases. 30th ed. Elk Grove Village, IL: American Academy of Pediatrics; 2015. p. 667-76.

25. Tischer CG, Hohmann C, Thiering E, Herbarth O, Muller A, Henderson J, et al. Meta-analysis of mould and dampness exposure on asthma and allergy in eight European birth cohorts: an ENRIECO initiative. Allergy. 2011;66(12):1570-9.

26. Jackson KD, Howie LD, Akinbami LJ. Trends in allergic conditions among children: United States, 1997-2011. NCHS Data Brief. 2013(121):1-8.

27. Alduraywish SA, Lodge CJ, Campbell B, Allen KJ, Erbas B, Lowe AJ, et al. The march from early life food sensitization to allergic disease: a systematic review and meta-analyses of birth cohort studies. Allergy. 2016;71(1):77-89.

28. Centers for Disease Control and Prevention. Summary Health Statistics: National Health Interview Survey. 2015. https://ftp.cdc.gov/pub/Health_Statistics/NCHS/NHIS/SHS/2015_SHS_Table_C-2.pdf. Accessed 22 December 2017.

29. Hosseini B, Berthon BS, Wark P, Wood LG. Effects of Fruit and Vegetable Consumption on Risk of Asthma, Wheezing and Immune Responses: A Systematic Review and Meta-Analysis. Nutrients. 2017;9(4).

30. Lorson BA, Melgar-Quinonez HR, Taylor CA. Correlates of fruit and vegetable intakes in US children. J Am Diet Assoc. 2009;109(3):474-8.

31. Mebrahtu TF, Feltbower RG, Greenwood DC, Parslow RC. Childhood body mass index and wheezing disorders: a systematic review and meta-analysis. Pediatr Allergy Immunol. 2015;26(1):62-72.

32. Ogden CL, Carroll MD, Lawman HG, Fryar CD, Kruszon-Moran D, Kit BK, et al. Trends in Obesity Prevalence Among Children and Adolescents in the United States, 1988-1994 Through 2013-2014. JAMA. 2016;315(21):2292-9.

33. Ogden CL, Carroll MD, Curtin LR, McDowell MA, Tabak CJ, Flegal KM. Prevalence of overweight and obesity in the United States, 1999-2004. JAMA. 2006;295(13):1549-55.

34. Liu L, Pan Y, Zhu Y, Song Y, Su X, Yang L, et al. Association between rhinovirus wheezing illness and the development of childhood asthma: a meta-analysis. BMJ Open. 2017;7(4):e013034.

35. Busse WW, Lemanske RF, Jr., Gern JE. Role of viral respiratory infections in asthma and asthma exacerbations. Lancet. 2010;376(9743):826-34.

36. van Aalderen WM. Childhood asthma: diagnosis and treatment. Scientifica (Cairo). 2012;2012:674204.

37. Bowatte G, Lodge C, Lowe AJ, Erbas B, Perret J, Abramson MJ, et al. The influence of childhood traffic-related air pollution exposure on asthma, allergy and sensitization: a systematic review and a meta-analysis of birth cohort studies. Allergy. 2015;70(3):245-56.

38. Boehmer TK, Foster SL, Henry JR, Woghiren-Akinnifesi EL, Yip FY, Centers for Disease C, et al. Residential proximity to major highways - United States, 2010. MMWR Suppl. 2013;62(3):46-50.

39. Lin W, Brunekreef B, Gehring U. Meta-analysis of the effects of indoor nitrogen dioxide and gas cooking on asthma and wheeze in children. Int J Epidemiol. 2013;42(6):1724-37.

40. Centers for Disease C, Prevention. Use of unvented residential heating appliances--United States, 1988-1994. MMWR Morb Mortal Wkly Rep. 1997;46(51):1221-4.

41. Chen C, Xun P, Tsinovoi C, He K. Accumulated evidence on Helicobacter pylori infection and the risk of asthma: A meta-analysis. Ann Allergy Asthma Immunol. 2017;119(2):137-45 e2.

42. Staat MA, Kruszon-Moran D, McQuillan GM, Kaslow RA. A population-based serologic survey of Helicobacter pylori infection in children and adolescents in the United States. J Infect Dis. 1996;174(5):1120-3.

43. Gunaratne AW, Makrides M, Collins CT. Maternal prenatal and/or postnatal n-3 long chain polyunsaturated fatty acids (LCPUFA) supplementation for preventing allergies in early childhood. Cochrane Database Syst Rev. 2015(7):CD010085.

44. Black LI, Clarke TC, Barnes PM, Stussman BJ, Nahin RL. Use of complementary health approaches among children aged 4-17 years in the United States: National Health Interview Survey, 2007-2012. Natl Health Stat Report. 2015(78):1-19.

45. Lodrup Carlsen KC, Roll S, Carlsen KH, Mowinckel P, Wijga AH, Brunekreef B, et al. Does pet ownership in infancy lead to asthma or allergy at school age? Pooled analysis of individual participant data from 11 European birth cohorts. PLoS One. 2012;7(8):e43214.

46. American Veterinary Medical Association. U.S. pet ownership and demographics sourcebook. Schaumburg, IL: American Veterinary Medical Association; 2012.

47. Lochte L, Nielsen KG, Petersen PE, Platts-Mills TA. Childhood asthma and physical activity: a systematic review with meta-analysis and Graphic Appraisal Tool for Epidemiology assessment. BMC Pediatr. 2016;16:50.

48. Troiano RP, Berrigan D, Dodd KW, Masse LC, Tilert T, McDowell M. Physical activity in the United States measured by accelerometer. Med Sci Sports Exerc. 2008;40(1):181-8.

49. Elazab N, Mendy A, Gasana J, Vieira ER, Quizon A, Forno E. Probiotic administration in early life, atopy, and asthma: a meta-analysis of clinical trials. Pediatrics. 2013;132(3):e666-76.

50. Tinuoye O, Pell JP, Mackay DF. Meta-analysis of the association between secondhand smoke exposure and physician-diagnosed childhood asthma. Nicotine Tob Res. 2013;15(9):1475-83.

51. Homa DM, Neff LJ, King BA, Caraballo RS, Bunnell RE, Babb SD, et al. Vital signs: disparities in nonsmokers' exposure to secondhand smoke--United States, 1999-2012. MMWR Morb Mortal Wkly Rep. 2015;64(4):103-8.
